# Clinical and Biological Characteristics of Medullary and Extramedullary Plasma Cell Dyscrasias

**DOI:** 10.3390/biology10070629

**Published:** 2021-07-06

**Authors:** Snjezana Janjetovic, Philipp Lohneis, Axel Nogai, Derya Balci, Leo Rasche, Doris Jähne, Carsten Bokemeyer, Georgia Schilling, Igor Wolfgang Blau, Martin Schmidt-Hieber

**Affiliations:** 1 Department of Oncology, Hematology and Bone Marrow Transplantation with Section Pneumology, University Clinic Hamburg-Eppendorf, 20251 Hamburg, Germany; s.janjetovic@uke.de (S.J.); cbokemeyer@uke.de (C.B.); g.schilling@asklepios.com (G.S.); 2 Clinic of Hematology and Stem Cell Transplantation, HELIOS Clinic Berlin-Buch, 13125 Berlin, Germany; 3 Institute of Pathology, Charité University Medicine Berlin, 10117 Berlin, Germany; philipp.lohneis@uk-koeln.de; 4 Institute of Pathology, University of Cologne, 50923 Cologne, Germany; 5 Clinic of Hematology, Oncology and Tumor Immunology, Campus Benjamin Franklin, Charité University Medicine Berlin, 12203 Berlin, Germany; axel.nogai@charite.de (A.N.); balci.der@gmail.com (D.B.); igor.blau@charite.de (I.W.B.); 6 St. Joseph Hospital Berlin-Tempelhof, 12101 Berlin, Germany; 7 Department of Internal Medicine II, University Hospital Würzburg, 97080 Würzburg, Germany; rasche_l@ukw.de; 8 Institute of Pathology, HELIOS Clinic Berlin-Zehlendorf, 14165 Berlin, Germany; doris.jaehne@helios-gesundheit.de; 9 Department of Hematology, Oncology, Palliative Care and Rheumatology, Asklepios Hospital Altona, Asklepios Tumorzentrum, 22763 Hamburg, Germany; 10 Clinic of Hematology, Oncology and Tumor Immunology, Campus Virchow Klinikum, Charité University Medicine Berlin, 10117 Berlin, Germany; 11 Clinic of Hematology and Oncology, Carl-Thiem-Klinikum, 03048 Cottbus, Germany

**Keywords:** plasma cell disorder, multiple myeloma, extramedullary, immunohistochemistry, cytogenetics

## Abstract

**Simple Summary:**

Extramedullary disease can occur either in multiple myeloma at the initial diagnosis or relapse or as primary extramedullary plasmocytoma/solitary osseous plasmocytoma. The exact molecular mechanisms underlying extramedullary spread of clonal plasma cells are not fully understood. The aim of our study was to assess further insights into clinical and biological characteristics of different types of extramedullary plasma cell disorders. We show that expression profiles of molecules involved in homing and cytogenetic abnormalities differ between various types of plasma cell dyscrasias, indicating the contrasting biology of these diseases.

**Abstract:**

*Background:* Extramedullary plasma cell (PC) disorders may occur as extramedullary disease in multiple myeloma (MM-EMD) or as primary extramedullary plasmocytoma (pEMP)/solitary osseous plasmocytoma (SOP). In this study, we aimed to obtain insights into the molecular mechanisms of extramedullary spread of clonal PC. *Methods:* Clinical and biological characteristics of 87 patients with MM-EMD (*n* = 49), pEMP/SOP (*n* = 20) and classical MM (*n* = 18) were analyzed by using immunohistochemistry (CXCR4, CD31, CD44 and CD81 staining) and cytoplasmic immunoglobulin staining combined with fluorescence *in situ* hybridization (cIg-FISH). *Results:* High expression of CD44, a cell-surface glycoprotein involved in cell-cell interactions, was significantly enriched in MM-EMD (90%) *vs*. pEMP/SOP (27%) or classical MM (33%) (*p* < 0.001). In addition, 1q21 amplification by clonal PC occurred at a similar frequency of MM-EMD (33%), pEMP/SOP (57%) and classical MM (44%). Conversely, del(17p13), t(4;14) and t(14;16) were completely absent in pEMP/SOP. Besides this, 1q21 amplification was identified in 64% of not paraskeletal samples from MM-EMD or pEMP compared to 9% of SOP or paraskeletal MM-EMD/pEMP and 44% of classical MM samples, respectively (*p* = 0.02). *Conclusion:* Expression of molecules involved in homing and cytogenetic aberrations differ between MM with or without EMD and pEMP/SOP.

## 1. Introduction

Extramedullary plasma cell (PC) dyscrasias may occur either in multiple myeloma (MM) as an extramedullary disease (MM-EMD) or as primary extramedullary plasmocytoma (pEMP)/solitary osseous plasmocytoma (SOP) in the absence of MM-defining criteria [1,2,3]. MM-EMD could develop initially at the diagnosis of MM or at the time of relapse/disease progression with a frequency of around 5–25% [3,4,5]. Extramedullary PC disorders are characterized by the ability of clonal PC (cPC) to become independent from the bone marrow (BM) microenvironment and infiltrate other organs and/or circulate freely in the blood [6]. pEMP/SOP generally has a favorable prognosis with a 10-year survival rate of around 75% [2,7]. In contrast, MM-EMD defines high-risk as a poor prognosis and median overall survival (OS) of around 2–3 years [4,5].

The exact molecular mechanisms underlying extramedullary spread of cPC have not yet been fully defined. There are different molecular changes on the PC membrane that allow them to escape the BM. For example, CXCR4 surface molecule, which is important in hematopoietic stem cell and PC homing to the BM, is downregulated on cPC as they become extramedullar [1,8]. However, conflicting data were shown for the expression of cellular adhesion membrane glycoprotein CD56. While Dahl *et al*. showed complete CD56 downregulation by cPC in EMD [9], Weinstock *et al*. found CD56 positivity in a subset of patients [10]. In contrast, the adhesion molecule CD44, which is involved in a variety of biological processes including tumor metastasis, is shown to be upregulated in cPC in extramedullary disease [9,10]. Furthermore, the expression of CD31 in cPC has been controversially discussed [11,12], and its role in MM progression has not yet been fully determined. CD81 is a cell surface protein, which is evolved in cell motility, signaling and BM homing. Few studies focused on the pathophysiological role of CD81 expression in patients with PC disorders [13,14]. It has been suggested that CD81 expression inhibits migration and invasion of cPC [15]. This statement was confirmed by Paiva *et al*. by showing CD81 downregulation on circulating *vs*. medullary cPC [13]. Moreover, Lou *et al*. showed absence of CD81 expression by cPC in patients with PC leukemia using immunohistochemistry (IHC) [16]. In summary, the CD81 expression status may play a role in disease progression and extramedullary spread of cPC disorders. 

Moreover, cytogenetic alterations have an important prognostic impact in MM. Del(13q), t(4;14) and del(17p) are the most common cytogenetic abnormalities in cPC derived from BM of MM patients developing MM-EMD [10,17]. However, these studies contained a rather small number of patients. Furthermore, there are only few studies on cytogenetic aberrations in MM-EMD or pEMP/SOP [18,19,20]. The most common cytogenetic aberration in MM-EMD is 1q21 gain followed by del(13q) or del(17p) and C-*MYC* amplification [18,19], while in pEMP del(13q) and rearrangements of immunoglobulin heavy chain gene locus at 14q32 are prevailing [20]. Moreover, the proliferation marker Ki-67 as well as nuclear accumulation of p53 were shown to be significantly increased in MM-EMD compared to BM cPC [21].

Thus, EMD remains challenging from a scientific perspective. To obtain further insights into clinical and biological characteristics of MM with or without EMD *vs.* pEMP/SOP we analyzed 87 patients with different PC disorders by using IHC and cytoplasmic immunoglobulin staining combined with fluorescence *in situ* hybridization (cIg-FISH). 

## 2. Results 

### 2.1. Patients

In total 87 patients were analyzed: 67 (77%) had MM and 20 (23%) had pEMP (*n* = 13) or SOP (*n* = 7). Among patients with MM, 18 (27%) had only medullary/osseous involvement (i.e., classical MM without EMD) at all investigation time points, while the remaining 49 (73%) patients presented with EMD either at the diagnosis (*n* = 21) or at the relapse/disease progression (*n* = 28). In patients with exclusively classical medullary/osseous MM, samples for IHC and cytogenetic studies were obtained from biopsies of focal osseous MM manifestations in 12 of the 18 patients (67%), and medullary biopsies were analyzed in the remaining 6 patients (33%).

**Figure 1:**
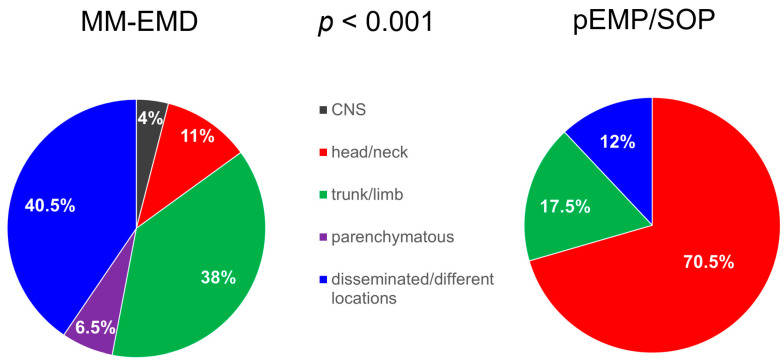
Distribution of locations of MM-EMD (*n* = 49) *vs.* pEMP/SOP (*n* = 20). Shown are percentages of patients. MM-EMD multiple myeloma with extramedullary disease; pEMP/SOP primary extramedullary plasmocytoma/solitary osseous plasmocytoma; CNS central nervous system.

In addition, 52% of MM-EMD tissue samples for IHC and/or cytogenetic studies were classified as paraskeletal, while this was the case only for 17% of pEMP samples (*p* = 0.08). Patient characteristics and distribution of locations of MM-EMD *vs*. pEMP/SOP are summarized in Table 1 and Figure 1. 

### 2.2. Immunohistochemistry 

**Figure 2:**
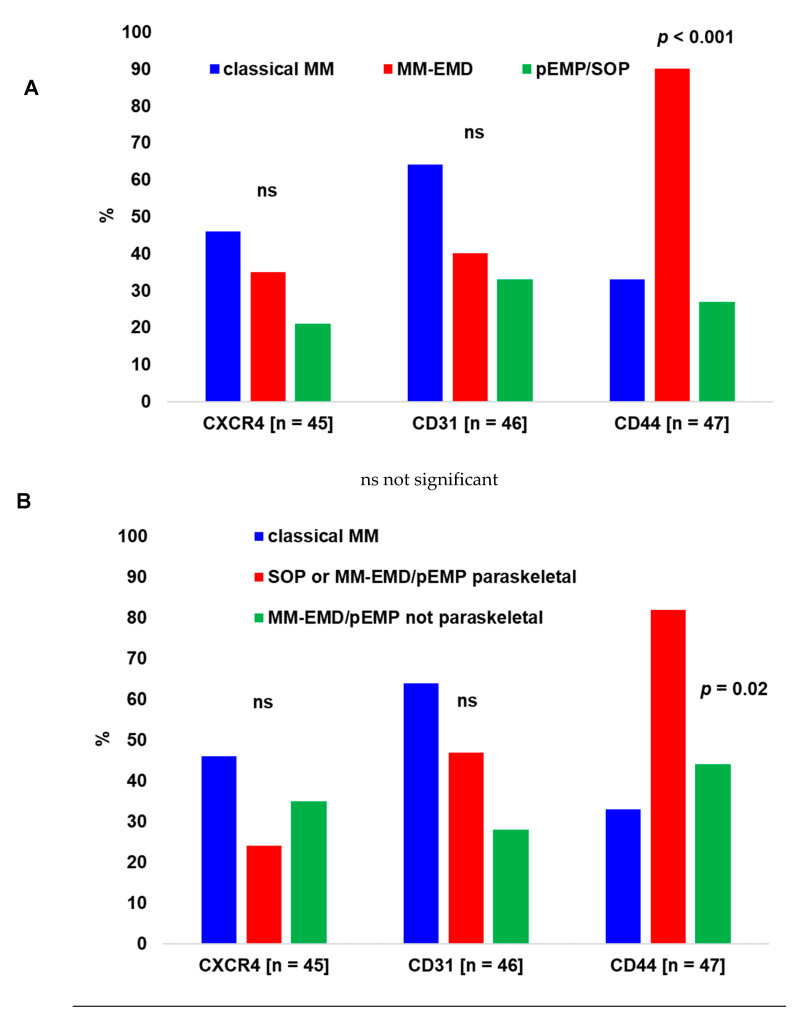

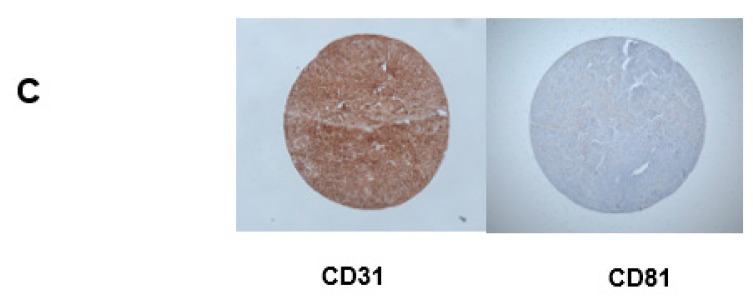
Expression of CXCR4, CD31, CD44 and CD81 by cPC. (**A**) Classical (medullary/osseous) MM *vs.* MM-EMD *vs*. pEMP/SOP. CD81 expression was not identified in any of 42 studied samples (not shown). (**B**) Classical MM *vs*. SOP or MM-EMD/pEMP paraskeletal *vs.* MM-EMD/pEMP not paraskeletal. Shown are percentages of samples with a moderate or strong expression intensity (*vs*. negativity or low expression) from tested samples. (**C**) Representative immunohistochemistry study on tissue microarrays (TMA) showing strong CD31 expression of cPC (left, buccal MM-EMD) and lack of CD81 expression (right, MM-EMD with skin infiltration).

Moderate/strong expression of CXCR4, CD31, CD44 and CD81 by cPC was identified in 15/45 (33%), 20/46 (43%), 26/47 (55%) and 0/42 (0%) of the samples respectively. The expression status of tested antigens by cPC for different patient groups is summarized in Figure 2. 

cPC from MM-EMD showed significantly more frequent moderate/strong CD44 positivity (90%, compared to weak expression/negativity) than those from classical MM (33%) or pEMP/SOP (27%) (*p* < 0.001, Figure 2A). If analyzing only patients with pEMP (i.e., excluding SOP) treated exclusively locally, moderate/strong expression of CXCR4, CD31 and CD44 was observed in 1/9 (11%), 4/10 (40%) and 1/10 (10%) of samples. Moderate/strong CD44 expression was also more prevalent in SOP or MM-EMD/pEMP paraskeletal (82%) compared to MM-EMD/pEMP not paraskeletal (44%) or classical medullary/osseous MM (33%) (*p* = 0.02, Figure 2B). Finally, moderate/strong CD44 expression was less frequent in MM-EMD or pEMP/SOP originating from the head/neck region (5/14 samples, 36%) vs. other regions (16/19 samples, 84%) (*p* = 0.009). However, distribution of locations of extramedullary cPC disease and CD44 expression status did not show a significant correlation when analyzing patients with MM-EMD *vs*. pEMP/SOP separately (data not shown).

### 2.3. Cytogenetics

At least one of the tested cytogenetic aberrations was observed in 77% of the samples while high-risk cytogenetics were identified in 29%. In summary, del(17p13), del(13q14), amplification, t(4;14), t(11;14), t(14;16) and 1q21 amplification was observed in 19%, 35%, 21%, 11%, 10%, 0% and 41% of the analyzed samples, respectively. 

**Figure 3:**
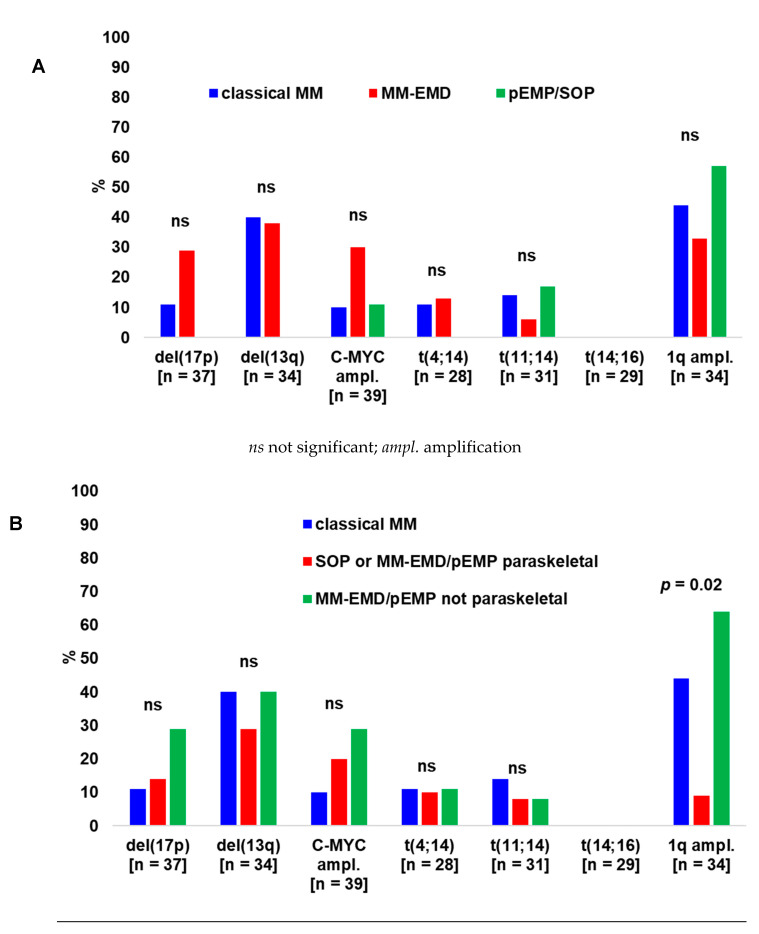

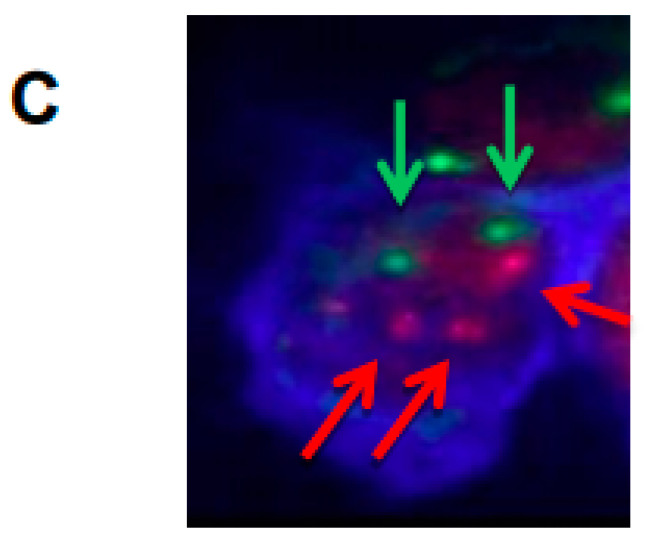
Cytogenetic aberrations of cPC. (**A**) Classical (medullary/osseous) MM *vs.* MM-EMD *vs.* pEMP/SOP. (**B**) Classical MM *vs*. SOP or MM-EMD/pEMP paraskeletal *vs.* MM-EMD/pEMP not paraskeletal. Shown are percentages of patients with a distinct cytogenetic aberration (from evaluated patients). (**C**) Representative cIg-FISH study showing *C-MYC* amplification in a patient with MM-EMD (skin infiltration). Amplified signals for *C-MYC* (red arrows) and two signals for p7t1 (centromere 7, green arrows) are demonstrated. Blue color stains intracytoplasmic light chains.

The frequencies of cytogenetic alterations in different patient groups are summarized in Figure 3. Del(17p13), del(13q14), t(4;14) and t(14;16) were not detected in any pEMP/SOP sample. Conversely, 1q21 amplification was identified in 57% of pEMP/SOP samples compared to 33% of MM-EMD and 44% of classical MM samples (*p* = 0.54, Figure 3A). Besides this, 1q21 amplification was significantly more frequent in MM-EMD/pEMP not paraskeletal (64%) compared to SOP or MM-EMD/pEMP paraskeletal (9%) or classical MM (44%) (*p* = 0.02, Figure 3B).

*C-MYC* amplification was significantly (*p* = 0.03) more frequent among patients with del(17p13) (4/7 patients, 57%) than in patients without this aberration (4/29 patients, 14%). Additionally, t(4;14) occurred more often (*p* = 0.04) among patients with del(13q14) (3/10 patients, 30%) compared to those without del(13q14) (0/17 patients, 0%). 

### 2.4. Treatment and Outcome

Patients with MM (65 evaluable with respect to treatment) received at diagnosis or at relapse/disease progression proteasome inhibitors (PI, *n* = 38, 58%), immunomodulatory imide drugs (IMiDs, *n* = 27, 42%) and/or autologous (*n* = 38, 58%) or allogeneic (*n* = 10, 15%) hematopoietic stem cell transplantation. 22 patients (34%) received neither PI nor IMiDs. Both PI and IMiDs were administered to 22 patients (34%). 

Primary treatment of patients with pEMP/SOP (*n* = 20) at diagnosis included excision/resection (*n* = 15, 75%) and/or irradiation (*n* = 12, 60%). Of these 20 patients, 11 (55%) showed disease relapse/progression, either exclusively locally and extramedullary (*n* = 4, 20%) or systemically, mainly with medullary involvement (*n* = 7, 35%). All 4 patients with only focal extramedullary relapse were treated locally again (resection and/or irradiation) while all 7 patients with relapse/progression to MM (or plasmablastic lymphoma in one patient) were treated systemically. 

Disease progression/death was more frequent among patients with moderate/strong CD44 expression by cPC (23/24 patients, 96%) compared to patients with low CD44 expression or its negativity (11/19 patients, 58%) (*p* = 0.006). Moreover, moderate or strong CXCR4 expression of cPC was associated with a significantly increased mortality rate (54% *vs*. 20%, *p* = 0.04). 

No other significant associations were observed between the expression status of different antigens (or presence of cytogenetic aberrations) and the rate of disease progression or relapse/death (or death only), respectively (data not shown). 

**Figure 4:**
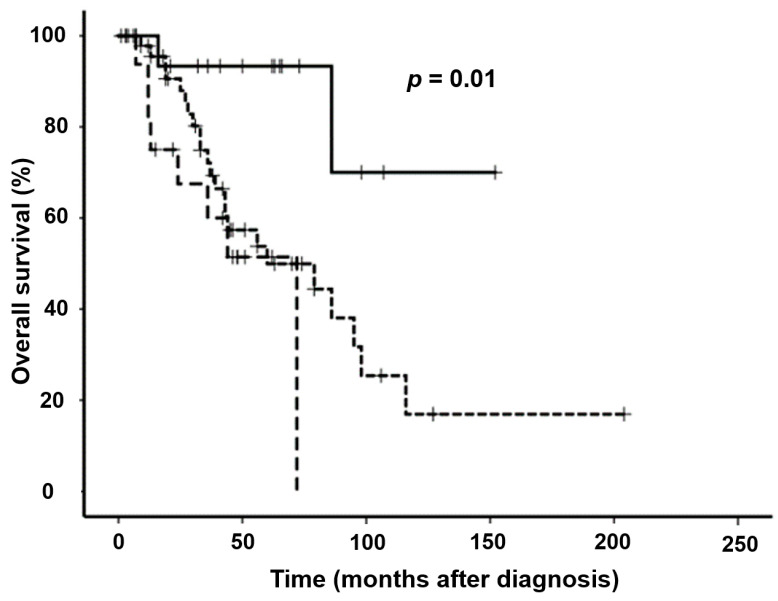
Overall survival of patients with MM with (– –) or without EMD (--) at diagnosis *vs.* pEMP/SOP (──). Shown are Kaplan–Meier estimates.

The median OS was significantly longer in patients with pEMP/SOP (not reached) than in patients with MM in conjunction with (72 months, 95%-CI not given) or without EMD at diagnosis (60 months, 95%-CI 14–106 months) (*p* = 0.01, Figure 4).

## 3. Discussion

Patients with MM-EMD mainly showed disseminated extramedullary disease (41%) or manifestations in the trunk or in the limbs (38%). The head/neck region was mostly affected in patients with pEMP/SOP (71%). A similar distribution of MM-EMD *vs*. pEMP/SOP was reported by others previously [1,2,3,5,10].

The precise mechanisms by which cPC escape the BM and became independent from the BM microenvironment are not fully clarified. One possible mechanism is alterations in the expression profile of surface adhesion molecules on cPC [1,6,22,23]. An increased expression of CD44 (or differential expression of variant isoforms such as CD44v3 or v6) in cPC of MM-EMD *vs*. the BM was already shown by some authors [9,10,22,23]. However, little is known about the significance of CD44 expression in pEMP/SOP [24]. In our study, we found significantly higher CD44 expression in cPC derived from MM-EMD compared to those from classical MM or pEMP/SOP. Furthermore, moderate/strong CD44 expression was also more frequent in SOP or MM-EMD/pEMP paraskeletal comparing to the cPC from classical MM or MM-EMD/pEMP not paraskeletal. Taken together, our findings confirm that increased CD44 expression plays a role in extramedullary cPC spread in MM [9]. Furthermore, our data led to assume that CD44 expression likely plays no role in extramedullary cPC spread in pEMP/SOP. Finally, our observations indicate that the pathomechanisms of extramedullary spread differ between cPC manifestations adjacent or non-adjacent to the bone. 

CD44v9 isoform expression has been associated with advanced Durie & Salmon stage in addition to IgA subtype and progressive disease [22]. Disease progression/death was also more frequent in patients with moderate/strong CD44 expression by cPC (96%) compared to those with low expression or negativity (58%) (*p* = 0.006) in our study. However, there was no significant association with IgA isotype or Durie & Salmon stage. Moreover, others found a correlation between chromosome 13q losses and CD44v6 expression [23]. In the present study, del(13q14) was observed in a similar frequency of samples with moderate/strong CD44 expression (32%) compared to those with CD44 low expression or negativity (33%). The reason for this discrepancy might be explained by the fact that we investigated standard CD44 and mainly included extramedullary disease while Liebisch *et al.* included monoclonal gammopathy of undetermined significance (MGUS), medullary MM and PC leukemia [23]. 

Finally, we observed lower CD44 expression intensities of cPC from extramedullary lesions from the head/neck region compared to other extramedullary locations. However, distribution of locations of extramedullary cPC disease had no significant impact on CD44 expression intensities if analyzing MM-EMD *vs*. pEMP/SOP separately. Besides this, CD44 expression intensities were significantly higher in MM-EMD compared to pEMP/SOP, as already specified above. Taken together, the different distribution of locations of extramedullary manifestations in MM-EMD *vs*. pEMP/SOP likely explains the observation that extramedullary cPC manifestations from the head/neck region had lower CD44 expression intensities than cPC originating from other regions. 

CD31 was shown to be expressed by all reactive PC and occasionally in pEMP [11,12]. While one study reported complete CD31 negativity in both medullary MM and MM-EMD [11], another study described 93% of PC myeloma and 80% of plasmablastic variants of myelomas to be CD31 positive [12]. We found no statistical difference in the frequency of moderate/strong CD31 expression between samples from MM-EMD (40%), classical MM (64%) and pEMP/SOP (33%). However, the percentage of samples with moderate/strong CD31 expression gradually decreased from cPC deriving from classical MM, MM-EMD and pEMP/SOP (and from classical MM to SOP or extramedullary manifestations paraskeletal *vs*. not paraskeletal). These findings indicate that CD31, an important vascular cell adhesion molecule, may play a role in extramedullary cPC spread.

CXCR4 expression intensities were also lower in extramedullary manifestations compared to classical MM in the present study, as already published [1] suggesting that CXCR4 losses may also play a role in cPC extramedullary spread. The impact of CD81 expression in the pathogenesis of PC disorders has been analyzed by different authors [13,14]. Using multiparameter flow cytometry, CD81 expression was identified in 45% of MM samples [13]. Lou *et al*. used CD81 staining by IHC in patients with different hematological malignancies, including PC disorders, and found that only 13% of MM BM samples but 0 of 10 samples from PC leukemia were CD81 positive [16]. Our analyses did not show any CD81 expression, suggesting that this marker probably has no significant role in MM-EMD or pEMP/SOP pathogenesis. 

The most frequent cytogenetic aberration in our study was 1q21 amplification, which was detected in a similar frequency of MM with (33%) or without EMD (44%) compared to pEMP/SOP (57%). 

The high frequency of 1q21 amplification (4/5 patients, 80%) was also found when analyzing only patients with pEMP treated solely by excision/resection and/or irradiation. Beside this, 1q21 amplification was significantly more frequent in extramedullary manifestations not paraskeletal compared to SOP or MM-EMD/pEMP paraskeletal or classical medullary/osseous MM. Contrasting 1q21 amplification, the high-risk aberrations del(17p13), t(4;14) and t(14;16) were not detected in any sample from pEMP/SOP in the present study. Taken together, our data suggest that 1q21 amplification occurs frequently in pEMP/SOP besides MM-EMD. Moreover, there was a trend towards a higher prevalence of del(17p13) and *C-MYC* amplification in extramedullary manifestations not paraskeletal compared to SOP or MM-EMD/pEMP paraskeletal or classical MM. 

As expected, patients with pEMP/SOP had a significantly longer OS than patients with MM with or without EMD at diagnosis with a 12-year estimated survival probability of around 75%, in concordance with published data [2,7].

## 4. Methods

### 4.1. Patients

Patients with MM (with or without EMD) or pEMP/SOP were identified from a database from the Institute of Pathology, Charité University Medicine Berlin or by patient chart review at other participating centers (HELIOS Clinic Berlin-Buch, Berlin; University Hospital Würzburg, Würzburg and University Clinic Hamburg Eppendorf, Hamburg, Germany). Important clinical characteristics were analyzed retrospectively by using patient chart review and/or electronic databases. 

Routine diagnostics (e.g., imaging, bone marrow analysis) and treatment of PC disorders followed local standards. Specific IHC studies included determination of the CXCR4, CD31, CD44 and CD81 expression status of cPC and were done centrally at the Institute of Pathology, Charité University Medicine Berlin. Identification of del(17p13), del(13q14), *C-MYC* amplification, t(4;14), t(11;14), t(14;16) and 1q21 amplification by cPC were done by cIg-FISH studies at the University Hospital Hamburg Eppendorf, Hamburg, Germany. IHC and cIg-FISH analyses were performed on formalin-fixed tissue sections or tissue microarrays (TMAs) of MM-EMD, pEMP/SOP and classical MM. This study was approved by the ethics committee and conducted in accordance with the Helsinki declaration. 

### 4.2. Definitions and Statistics

Modified International Myeloma Working Group (IMWG) criteria were used to classify different PC disorders, taking into account that imaging (e.g., computed tomography and magnetic resonance imaging) were not universally used in this study [25]. Besides this, BM analyses were not done in all patients with small focal pEMP/SOP, usually involving the head/neck region. Thus, the category `solitary plasmocytoma with minimal marrow involvement´ introduced in the IMWG 2014 classification is not represented in the present study [25]. Presence of extramedullary disease was histologically confirmed by biopsy in 45/48 patients (94%) with MM-EMD and 19/20 patients (95%) with pEMP/SOP. Presence of extramedullary involvement was diagnosed by imaging in the remaining 4 patients. cPC samples showing del(17p13), t(4;14) or t(14;16) were classified as chromosomal high-risk disease [26]. OS was defined as the time from diagnosis to death or last follow-up, respectively. Comparison of categorical variables was done using the Fisher’s exact or the Chi square test, whereas the Kruskal-Wallis test was used to compare continuous variables. The Kaplan–Meier method and the log-rank test were applied to compare OS among different patient groups. *P* values <0.05 were considered to be statistically significant. All statistical analyses were performed with the SPSS software, version 26.0 (Chicago, IL, USA). 

### 4.3. Immunohistochemistry

Expression intensities of CXCR4, CD31, CD44 and CD81 were carried out on TMAs, whereas one or two tissue cores 1 mm in diameter were analyzed for each sample. TMA sections were deparaffinized and subjected to heat-induced epitope retrieval before incubation with primary antibodies using the automated Leica Bond Max™ system (Leica Microsystems, Wetzlar, Germany). Antibodies were directed against CXCR4 (polyclonal # PA3-305; Thermo Fisher Scientific, Waltham, MA, USA), CD31 (clone JC70A, Dako, Glostrup, Denmark), CD44 (clone DF14/85, Dako, Glostrup, Denmark) and CD81 (clone M38, Thermo Fisher Scientific, Waltham, MA, USA). The detection kit Bond Polymer Refine (Leica Microsystems, Nußloch, Germany) was used for visualization of bound antibodies. Tonsil tissue was used as a positive control. Staining intensity was semi-quantitatively assessed as negative *vs*. weak, moderate, and strong.

### 4.4. Cytogenetics

We combined FISH with stains for intracytoplasmic light chains (lambda or kappa) by fluorescence-labeled antibodies (cIg-FISH) as described previously [18]. At least 100 cells were scored per sample. The following commercially available DNA probes were used for the analyses: LSI Vysis LSI TP53 spectrum orange mapping at 17p13, LSI Vysis D13S319 spectrum orange mapping at 13q14.3, and LSI Vysis *C-MYC* which was used to detect *MYC* amplification but not rearrangements of this gene (all Abbott Diagnostics, IL, USA). A probe hybridizing to the centromere region of chromosome 7 (p7t1) served as control for detection of 17p13 and 13q14.3, respectively. We used the dual color translocation probes LSI IGH/FGFR3, LSI IGH/CCND1 and LSI IGH/MAF (all Abbott Diagnostics, IL, USA) to detect the translocations t(4;14), t(11;14) and t(14;16), respectively. Finally, 1q21 amplification was recorded if >3 copies per cell were present and identified by using the CKS1B/CDKN2C probe (Cytocell, Cambridge, United Kingdom). All probes were hybridized according to the manufacturer’s instructions. In total, four MM-EMD and three pEMP/SOP samples were analyzed on conventional tissue slides while the remaining probes were examined on TMAs. 

## 5. Conclusions

In summary, we show that expression profiles of molecules involved in homing and cytogenetic abnormalities differ between different types of PC dyscrasias. Del(17p13), t(4;14) and t(14;16) are virtually absent in pEMP/SOP. Conversely, 1q21 amplification seems to be present in similar proportion of samples from pEMP/SOP, MM-EMD, and classical MM. 

## Figures and Tables

**Table 1:** Patient characteristics. Shown are numbers (*n*) of evaluable patients and percentages (%) or median values with ranges and evaluated patients, respectively.

	MM without EMD (at Different Time Points Studied) (*n* = 18)	MM with EMD (at Any Studied Time Point) (*n* = 49)	pEMP/SOP (at Diagnosis) (*n* = 20)	*p*
Age, years (*n* = 87)	60 (44–89)	63 (40–84)	61 (19–76)	ns
Gender				ns
Male	11 (61%)	31 (69%)	12 (60%)	
Female	7 (39%)	14 (31%)	8 (40%)	
Isotype				ns
IgG	10 (59%)	26 (54%)	7 (44%)	
IgA	4 (23.5%)	14 (29%)	3 (19%)	
Light chain	3 (17.5%)	8 (17%)	6 (37%)	
Light chain type				ns
Kappa	11 (65%)	31 (66%)	14 (74%)	
Lambda	6 (35%)	16 (34%)	5 (26%)	
Durie & Salmon stage				ns
I	4 (25%)	6 (13.5%)	-	
II	2 (12.5%)	3 (7%)	-	
III	10 (62.5%)	35 (79.5%)	-	
A	13 (76.5%)	30 (88%)	-	
B	4 (23.5%)	4 (12%)	-	
Serum concentrations at diagnosis				
Creatinine	73 (41–487)	91 (50–200)	57 (47–84)	0.05
(µmol/L, *n* = 44)				
ß2 microglobulin	3.5 (1.4–10.8)	2.5 (1.4–13.1)	2.0 (1.1–7.2)	ns
(mg/L, *n* = 41)				
Calcium	2.3 (1.7–4.1)	2.3 (2.0–4.0)	2.4 (2.1–3.8)	ns
(mmol/L, *n* = 46)				
LDH (U/L, *n* = 43)	155 (83–271)	188 (80–3284)	165 (143–2244)	ns
% BM PC at diagnosis (MM, *n* = 44)	40 (10–95)	30 (0–100)	-	ns
% of KI-67+ cells (MM-EMD/pEMP/SOP samples, *n* = 30)	-	38 (5–90)	30 (5–100)	ns
Year of diagnosis (*n* = 87) *	2005	2008	2007	ns
	(1996–2009)	(1996–2013)	(1994–2011)	
Time of follow-up, months (*n* = 83)	49 (13–79)	35 (1–204)	50 (3–152)	ns
Mortality rate (*n*/evaluated patients)	9/18 (50%)	22/46 (48%)	2/19 (11%)	0.01

LDH lactate dehydrogenase; BM bone marrow; PC plasma cells; MM multiple myeloma; EMD extramedullary disease; pEMP/SOP primary extramedullary plasmocytoma/solitary osseous plasmocytoma; ns not significant. * biopsies for immunohistochemistry and fluorescence in situ hybridization studies were taken between 1994 and 2013 (median year 2007).

## Data Availability

Not applicable.
